# Differential analysis of gut microbiota between captive and wild forest musk deer (*Moschus berezovskii*) based on 16S rRNA sequencing

**DOI:** 10.3389/fvets.2026.1824527

**Published:** 2026-06-04

**Authors:** Min Lu, Liwen Zhu, Kun Dai, Yanhong Wang, Song Yao, Shaobin Li, Zhaohui Xie

**Affiliations:** 1 School of Life Sciences and Engineering, Henan University of Urban Construction, Pingdingshan, China; 2 School of Life Science, Yangtze University, Jingzhou, China; 3 Neixiang Management Bureau of Baotianman National Nature Reserve, Neixiang, Henan, China; 4 College of Life Sciences, South-Central Minzu University, Wuhan, China

**Keywords:** 16S rRNA sequencing, captive, forest musk deer, gut microbiota, wild

## Abstract

Forest musk deer (*Moschus berezovskii*) is a globally endangered species, and its conservation has long been a matter of concern. Wild populations are scarce, while artificially captive populations are also constrained by health issues such as digestive system diseases. To reveal the differences in gut microbiota between captive and wild forest musk deer from different geographical regions, fecal samples were collected from captive individuals in Nanyang, Henan (HN) and Gaoping, Shanxi (SX), as well as wild individuals in Baotianman, Henan (YS), with 5 samples per group. High-throughput 16S rRNA sequencing was employed to analyze the microbial community structure and function. The sequencing revealed Firmicutes, Bacteroidota, and Proteobacteria as the dominant phyla across all three groups, with Actinobacteriota exhibiting a significantly higher abundance in the YS wild group (11.13%) than in the HN (1.29%) captive and SX (6.12%) captive groups. There were no significant differences in *α*-diversity among the groups. However, *β*-diversity analysis (PCoA and NMDS) indicated a clear separation in microbial community structure between captive and wild groups, with some individuals in the SX captive group clustering with the wild group. LEfSe analysis identified 36 differential biomarkers: the YS wild group was enriched in genera including *Bacillus*, *Arthrobacter*, and *Microbacterium*, whereas the HN captive group was enriched in *Bacteroides*, *Clostridium*, and *Eubacterium*, while the SX captive group was enriched in *Skermanella* (genus) and Cytophagales (order). Functional prediction analysis revealed that the gut microbiota of the wild group was significantly enriched in the pathways of xenobiotics biodegradation and metabolism as well as lipid metabolism, whereas the captive groups showed higher activity in the translation and nucleotide metabolism pathways. This study reveals the impacts of rearing methods and geographical factors on the gut microbial community structure and function of forest musk deer. These findings can serve as a theoretical foundation for promoting healthy breeding of captive populations and as a reference for evaluating the health status of wild populations.

## 1 Introduction

The forest musk deer (*Moschus berezovskii*) is a small, solitary cervid species inhabiting mountain forests in East Asia. Due to the illegal poaching of musk glands in males, its population has been declining steadily, and it is currently listed as an endangered species by the International Union for Conservation of Nature (IUCN) (1). Despite the long-standing reliance on its musk resources in traditional medicine and the perfume industry, the focus of current conservation efforts has shifted to integrating habitat conservation with artificial breeding, and exploring sustainable, non-invasive musk collection techniques to achieve a balance between ecological protection and resource utilization (2). However, digestive tract diseases are prevalent in captive forest musk deer (3, 4), and the underlying gut microbial mechanisms remain unclear, hindering the improvement of population health management strategies. The gut ecosystem is characterized by dynamic and reciprocal interactions between the host and bacteria (5). The complex microbial communities colonizing host animals, particularly the gut microbiota, can be regarded as a “functional extension system” (6), which plays a vital role in mediating key physiological processes such as nutrient metabolism, immune regulation, and environmental adaptation (7, 8). In ruminants, the efficient digestion of high-fiber diets relies on the synergistic interaction between rumen and gut microorganisms, such as the secretion of cellulases and the production of short-chain fatty acids (SCFAs) mediated by specific bacterial groups (9, 10).

Studies have shown that dietary simplification and spatial confinement under captive conditions can lead to reduced diversity and functional dysfunction of the gut microbiota in endangered species such as giant pandas (*Ailuropoda melanoleuca*) and snub-nosed monkeys (*Rhinopithecus brelichi*) (11, 12). The structure and function of the gut microbiota in captive forest musk deer vary with seasonal changes (13, 14). The *α*-diversity of the gut microbiota in forest musk deer is higher in the cold season than in the warm season, suggesting that seasonal alternation can affect the diversity of musk deer gut microbiota. This change is beneficial to their environmental adaptation and food digestion and metabolism (13). Recent studies on forest musk deer gut microbiota have focused on comparing wild populations from different regions with local captive individuals, revealing that wild groups enrich Pseudomonadota and lignin-degrading genera, while captive groups show increased Bacillota and pathogenic bacteria (15). Differences in the plant composition of the feed is likely to influence the community structure of the forest musk deer’s gut microbiota through varying nutrient inputs and secondary metabolite intake (16). However, the gut microbial divergence among captive populations from different geographical locations, and whether specific captive management can drive microbiota convergence to wild counterparts, remain unaddressed.

This study selected two representative captive populations of forest musk deer from Nanyang, Henan Province and Gaoping, Shanxi Province, as well as a wild population from the Baotianman National Nature Reserve in Henan Province, as the research subjects. Through a comparative design across multiple geographical populations and combined with 16S rRNA sequencing technology, the differences in community structure and functional adaptive differentiation of the gut microbiota between captive and wild forest musk deer were analyzed. This provides a scientific basis for the breeding management of captive forest musk deer.

## 2 Materials and methods

### 2.1 Sample collection

Sampling was conducted in April (spring), which is outside the peak musk secretion season of forest musk deer (17, 18). Fecal samples of captive forest musk deer were collected from two breeding farms: Nanyang City (HN, 33°21′N, 111°25′E, *n* = 5) in Henan Province and Gaoping City (SX, 35°45′N, 113°5′E, *n* = 5) in Shanxi Province, China. All forest musk deer were housed in semi-open enclosures with separate indoor pens and outdoor activity areas. The HN farm had brick-paved activity areas (15–20 m^2^) with a small vegetated section, whereas the SX farm had cement-paved activity areas (20–30 m^2^) with a larger soil area planted with trees, providing musk deer with greater access to natural soil and vegetation. The daily diet of captive forest musk deer consists of tender twigs, buds, and herbaceous plants from local vegetation, supplemented with a certain proportion of concentrated feed. All captive individuals (HN and SX groups) were clinically healthy, with no antibiotics or probiotics administered for at least 3 months prior to sampling. Enclosures were cleaned 1 day prior to sampling to ensure the collection of fresh morning feces. Fecal samples of wild forest musk deer were collected from the Baotianman National Nature Reserve in Henan Province (YS, 33°20′–33°36′N, 111°47′–112°04′E, *n* = 5). The survey was conducted using the transect method, with feces that appeared fresh in form and moist in surface collected along the survey route. Samples were collected at intervals ≥1,000 meters along transects, with distinct fecal morphology, size, and deposition sites to ensure independence, although molecular individual identification was not performed (the sampling sites were shown in Supplementary Figure S1).

The contamination-free central part of fresh feces was selected and placed into sterile sampling bags, immediately transported back to the laboratory within 4 h using a portable vehicle-mounted refrigerator at 4 °C, and then transferred to an ultra-low temperature refrigerator at −80 °C for long-term storage until use (detailed sample information can be found in Supplementary Table S1).

### 2.2 Genomic DNA extraction and PCR amplification

Genomic DNA was extracted from the samples using the MagPure Stool DNA KF Kit B (Magen, Guangzhou, China) in accordance with the manufacturer’s instructions. The DNA concentration and purity (A260/A280 ratio) were determined using a NanoDrop 2000 spectrophotometer (IMPLEN, Westlake Village, CA, United States). The integrity of the DNA was further assessed by 1% agarose gel electrophoresis.

Universal primers 338F (5’-ACTCCTACGGGAGGCAGCAG-3′) and 806R (5’-GGACTACHVGGGTWTCTAAT-3′), which are equipped with sequencing adapters, were used to amplify the V3–V4 hypervariable region of the bacterial 16S rRNA gene (19, 20). PCR reactions were performed using the TransStart Fastpfu DNA Polymerase kit (TransGen, Beijing, China). Three technical replicates were prepared for each sample, with a reaction mixture volume of 20 μL. The PCR program was as follows: pre-denaturation at 95 °C for 3 min; 30 cycles of denaturation at 95 °C for 30 s, annealing at 56 °C for 30 s, and extension at 72 °C for 45 s; and a final extension at 72 °C for 10 min. After pooling the three PCR replicates from the same sample, the mixture was subjected to 2% agarose gel electrophoresis for detection. The target bands were excised and purified using the AxyPrep DNA Gel Extraction Kit (AXYGEN, Hangzhou, China), and then eluted with Tris–HCl buffer.

### 2.3 Library construction and sequencing

The sequencing library was constructed using the VAHTS Universal DNA Library Prep kit for Illumina V3 (Vazyme, Nanjing, China). The workflow included DNA fragmentation, end repair, adapter ligation, as well as library enrichment and size selection. The constructed library was quantified using a Qubit® 3.0 Fluorometer, and the fragment size distribution was detected by an Agilent 2,100 Bioanalyzer. The quality-checked libraries were subjected to paired-end (PE) 250 bp sequencing on the Illumina NovaSeq 6,000 platform at Shanghai Yuanxin Biopharmaceutical Technology Co., Ltd. (Shanghai, China).

### 2.4 Bioinformatics analysis

#### 2.4.1 Data quality control

Raw Reads data were subjected to quality control as follows: data were demultiplexed based on sample-specific barcodes and primer sequences; FLASH software (v1.2.11) was used to assemble paired-end reads; Trimmomatic software (v0.39) was employed to filter low-quality sequences (quality score Q < 20, sequence length <50 bp) (21). Finally, Usearch software (v7.0.1090) was used to remove chimeric sequences by combining denoising (denovo) and reference-based approaches against the gold database, yielding high-quality clean tags for each sample (22, 23).

#### 2.4.2 OTU clustering and taxonomic analysis

All high-quality clean tags were clustered into Operational Taxonomic Units (OTUs) at a 97% similarity threshold using Usearch software (v7.0.1090). OTU-based approach was chosen to ensure methodological consistency and comparability with previous studies on forest musk deer gut microbiota that used the same methodology (13, 14). One representative sequence was selected from each OTU. The RDP Classifier bayesian algorithm (v2.2, confidence threshold = 0.7) was used for taxonomic annotation of OTU representative sequences (24). The species composition of each sample was analyzed at six taxonomic levels: phylum, class, order, family, genus, and species.

#### 2.4.3 Diversity analysis

*α*-diversity indices, including Observed species, Chao1, ACE, Shannon, and Simpson indices, were calculated using mothur software (v1.41.0) (25). The Kruskal-Wallis test (implemented in R software, v3.4.4) was used to compare differences among three groups, with a significance level set at *p* < 0.05. The adequacy of sequencing depth and sample size was assessed by plotting rarefaction curves and species accumulation curves. *β*-diversity distance matrices were calculated using the Bray-Curtis and Unweighted UniFrac distance algorithms. Principal Coordinates Analysis (PCoA) and Non-metric Multidimensional Scaling (NMDS) were performed using the “vegan” and “ggplot2” packages in R software (v3.4.4), and Adonis tests were conducted to evaluate the statistical significance of differences between groups.

#### 2.4.4 Differential species analysis

Linear discriminant analysis (LDA) was performed using LEfSe (v1.0) to identify biomarkers with significantly different abundance among three groups (26). The Kruskal-Wallis test was used to screen differentially abundant taxa between groups (*p* < 0.05). The LDA analysis was used to calculate the effect size of differences. Taxa with LDA score > 2 were considered differentially abundant. To identify taxa with larger effect sizes, a more stringent threshold of LDA > 3 was applied, and these were defined as major differential taxa (27, 28).

#### 2.4.5 Functional prediction analysis

The PICRUSt2 software (v2.5.2) was used to predict the metagenomic functional potential of the gut microbial community based on the OTU abundance table (29). The predicted gene families were mapped to the KEGG database to obtain pathway abundance information at different levels (Level 1, 2, and 3). The STAMP software (v2.1.3) was used to perform non-parametric tests (Kruskal-Wallis test) on the abundance of pathways at Level 2 between groups, and Dunn’s *post hoc* tests were used for pairwise comparisons. A *p*-value < 0.05 was considered statistically significant.

## 3 Results

### 3.1 OTU clustering and microbial community composition analysis

Fecal samples from 15 forest musk deer were subjected to sequencing, and a total of 1,422,996 high-quality sequences were obtained after quality control (detailed sequencing data are provided in Supplementary Table S2). Both the rarefaction curves and species accumulation curves tended to plateau, indicating that the sequencing depth was sufficient to cover the majority of microbial species in the samples (Supplementary Figure S2).

**Figure 1:**
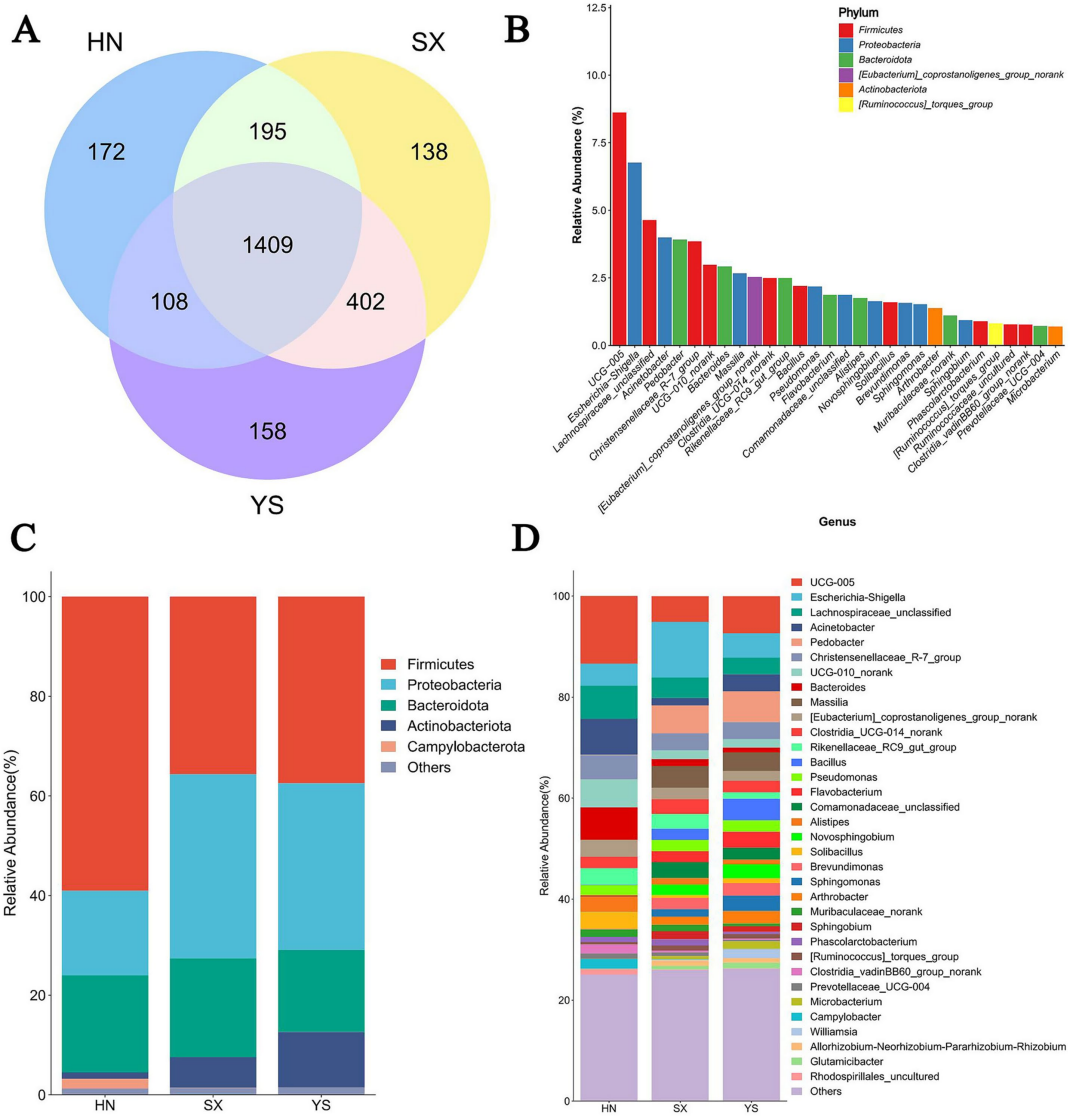
Clustering and composition of microbial community in feces of forest musk deer. **(A)** Venn diagram of OTU counts for three sample groups. **(B)** Bar chart of the top 30 dominant bacterial genera in relative abundance at the genus level for all samples (the same color indicates the same phylum). **(C)** Community composition at the phylum-level in the three sample groups. **(D)** Community composition at the genus-level in three sample groups.

At a 97% similarity threshold, all sequences were clustered into 2,582 Operational Taxonomic Units (OTUs). The Venn diagram analysis (Figure 1A) showed that the three groups (HN, SX, YS) shared 1,409 OTUs, accounting for 54.6% of the total OTUs, which constituted the core gut microbial community of forest musk deer. The HN, SX, and YS groups each had 172, 138, and 158 unique OTUs, respectively. All OTUs were annotated to 26 phyla, 53 classes, 138 orders, 275 families, and 646 genera. A bar chart was constructed by selecting the top 30 dominant genera with the highest relative abundance at the genus level across all samples (Figure 1B). Dominant genera such as *Lachnospiraceae_unclassified*, *Acinetobacter*, *Pedobacter*, *Christensenellaceae_R-7_group*, and *Bacteroides* formed the stable foundation of the forest musk deer gut microbiota.

At the phylum level (Figure 1C), Firmicutes, Bacteroidota, and Proteobacteria were the absolute dominant phyla shared by all three groups. Notably, the average abundance of Actinobacteriota in the YS wild group (11.13%) was significantly higher than that in the two captive groups (HN: 1.29%; SX: 6.12%). The HN captive group exhibited a community structure dominated by Firmicutes (59.01%), which was significantly higher than that in the SX captive group (35.61%) and YS wild group (37.45%). The SX captive group showed a more similar ratio of Firmicutes and Proteobacteria to the YS wild group.

At the genus level (Figure 1D), the dominant genera differed among the groups. In the HN captive group, the dominant genera were *UCG-005* (13.38%), *Acinetobacter* (7.12%), *Lachnospiraceae_unclassified* (6.57%), *Bacteroides* (6.40%), and *UCG-010 norank* (5.53%). In the SX captive group, the genus *Escherichia-Shigella* had the highest abundance (11.05%), followed by *Pedobacter* (5.51%), *UCG-005* (5.01%), *Massilia* (4.28%), and *Lachnospiraceae_unclassified* (4.03%). The dominant genera in the YS wild group included *UCG-005* (7.33%), *Pedobacter* (6.11%), *Escherichia-Shigella* (4.85%), *Bacillus* (4.26%), and *Massilia* (3.65%).

### 3.2 Microbial diversity analysis

**Figure 2:**
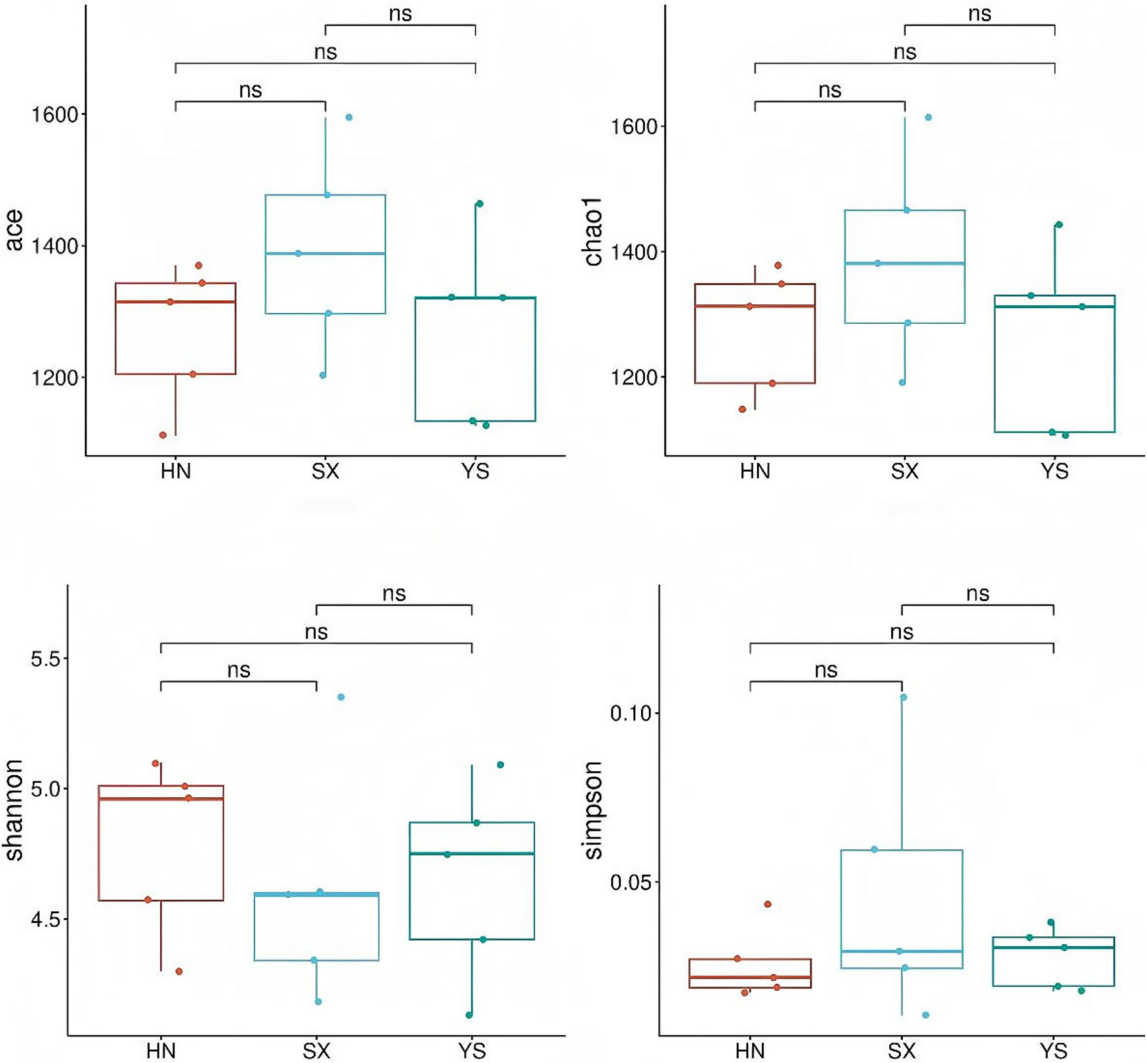
Comparison of *α*-diversity index of microbial community in feces of three groups of forest musk deer. Box plots show the ACE index (community richness), Chao1 index (community richness), Shannon index (community diversity), and Simpson index (community diversity), respectively. The horizontal line in the box represents the median, the box represents the interquartile range. NS indicates no significant difference between groups *P* > 0.05.

*α*-diversity analysis (Figure 2) showed that the Coverage index of all groups was above 99%, indicating that the sequencing depth was sufficient to reflect the true structure of the microbial community (α-diversity statistics in Supplementary Table S3). The SX captive group had slightly higher values of the ACE and Chao1 indices, suggesting that the microbial richness of the SX captive group was numerically higher. The HN captive group had a slightly higher Shannon index and the lowest Simpson index, indicating that its microbial community had higher diversity and more evenness. However, there were no significant differences in the ACE, Chao1, Shannon, and Simpson indices of the gut microbiota among the three groups (*p* > 0.05). This suggests that the captive versus wild environment, as well as the feeding conditions of different geographically captive populations, did not significantly affect the overall species richness and diversity level of the forest musk deer’s gut microbiota.

**Figure 3:**
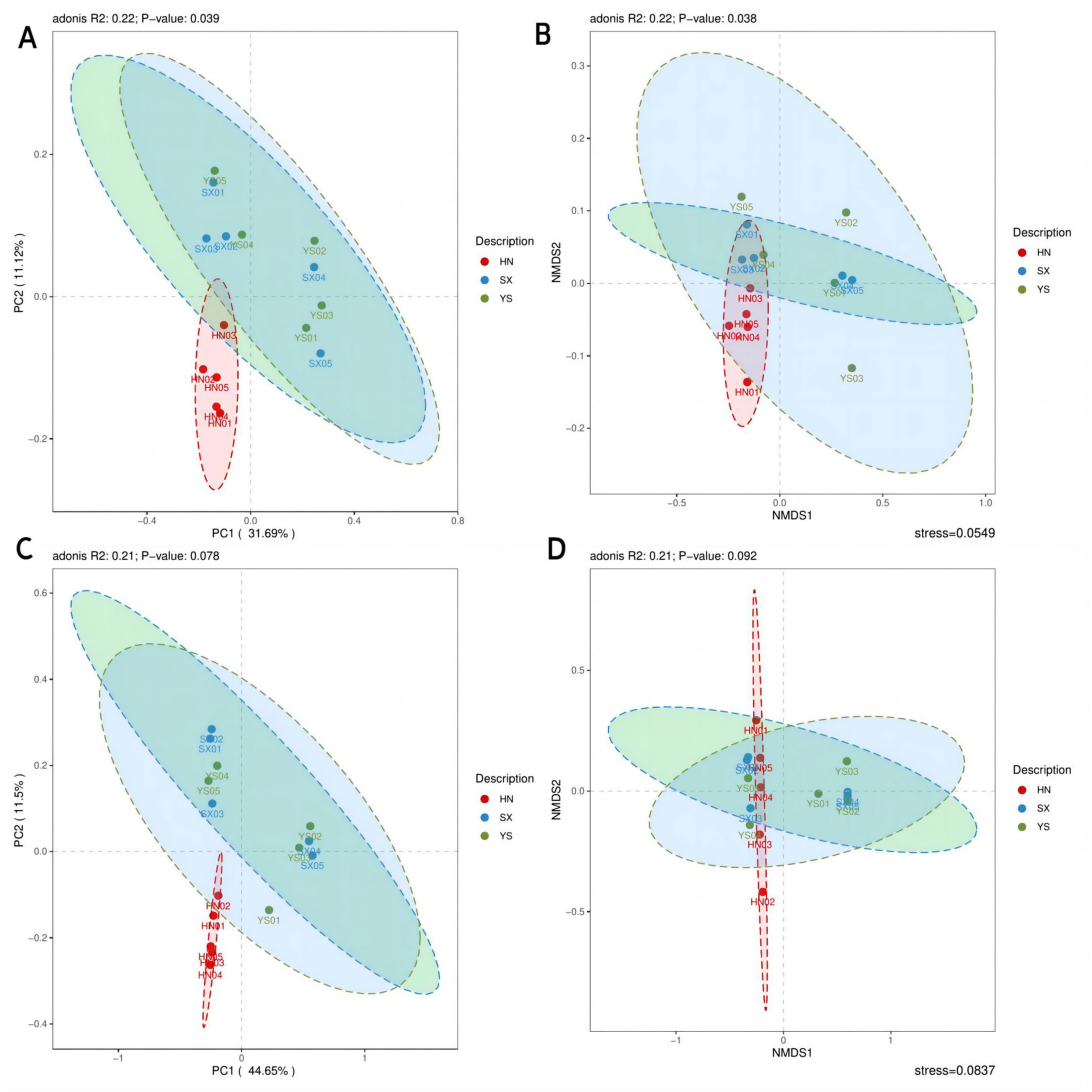
Analysis of *β*-diversity of microbial community in feces of three groups of forest musk deer. **(A,C)** Principal coordinate analysis (PCoA) based on unweighted UniFrac distance **(A)** and Bray-Curtis distance **(C)**; **(B,D)** Non-metric multidimensional scaling (NMDS) based on unweighted UniFrac distance **(B)** and Bray-Curtis distance **(D)**. Ellipses represent 95% confidence intervals, Adonis (PERMANOVA) was used to assess intergroup differences, and stress value reflects ordination reliability.

*β*-diversity analysis was performed using the Unweighted UniFrac distance and Bray-Curtis distance to reveal the structural differences in the gut microbiota of the three groups of forest musk deer (Figure 3). Unweighted UniFrac distance analysis (Figures 3A,B) revealed that the first and second principal components of PCoA explained 31.69 and 11.12% of the total microbial community variation, respectively. The microbial community structure of wild forest musk deer (YS) was most distinctly separated from the HN captive group, while the separation from the SX captive group was relatively weaker. Captive groups HN and SX also exhibited certain population specificity. NMDS analysis (stress = 0.0549) further verified this separation pattern, indicating that the ordination results were reliable. Adonis (PERMANOVA) test demonstrated that the structural differences in the gut microbiota among the three groups of forest musk deer were statistically significant (*R*^2^ = 0.22, *p* = 0.039). Bray-Curtis distance analysis (Figures 3C,D) showed that the first principal component of PCoA explained 44.65% of the microbial community variation. The between-group separation pattern was consistent with that observed in the Unweighted UniFrac analysis, but the degree of separation was slightly weaker. NMDS analysis (stress = 0.0837) also supported these results.

Combining the results of *α* and *β* diversity analyses, it was found that the overall diversity level of the gut microbiota of captive and wild forest musk deer was similar, but their species composition and structure differed significantly. The microbial community structure of captive forest musk deer from different geographical populations also exhibited geographical differentiation, which was mainly reflected at the level of species presence/absence, while the contribution of differences in microbial abundance was relatively small. The above results provide important insights for elucidating the shaping mechanism of the captive environment on the gut microbiota of forest musk deer and optimizing captive breeding management strategies.

**Figure 4:**
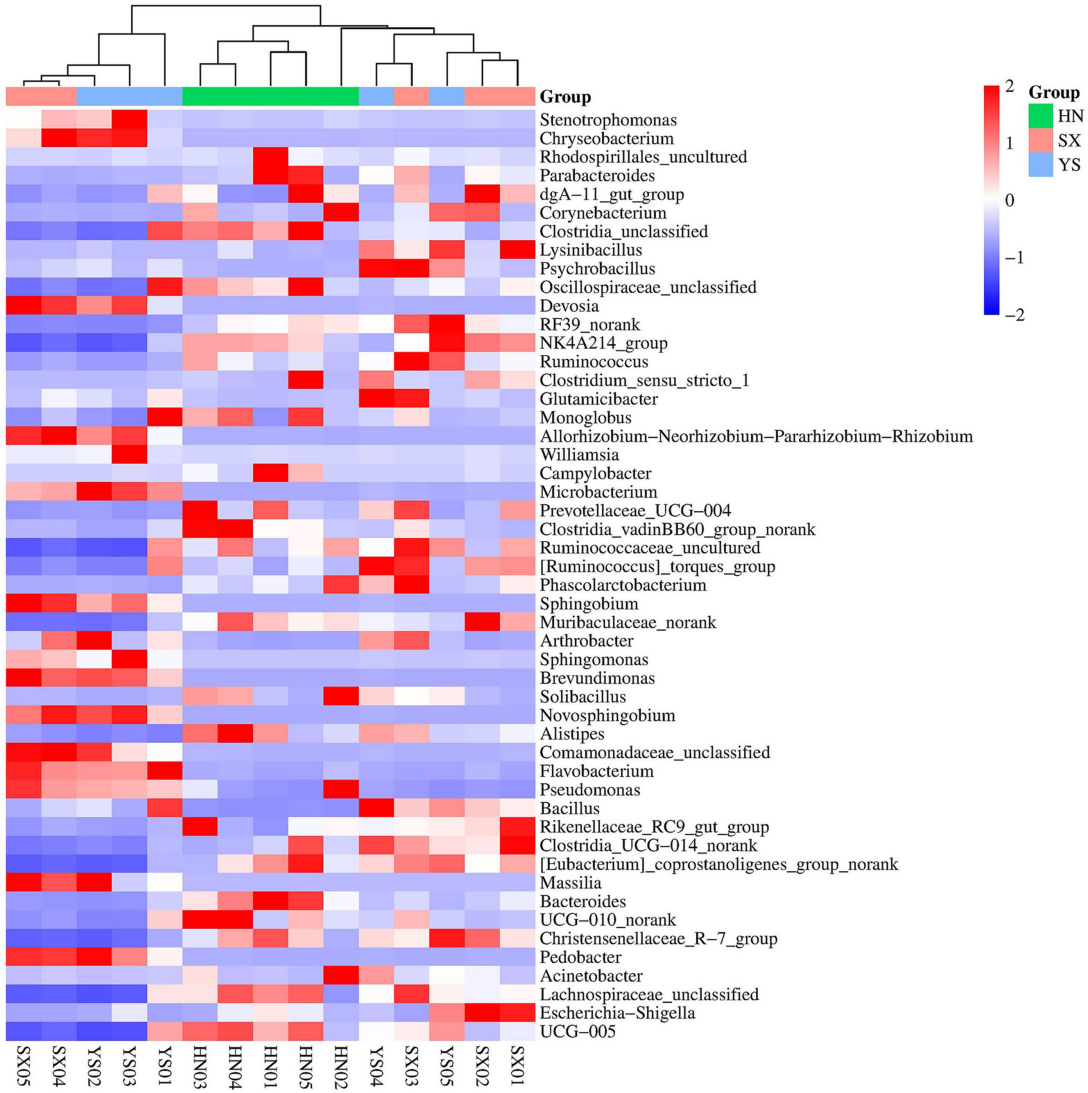
Cluster heat map of the top 50 microbial taxa in the feces of three groups of forest musk deer at the genus level. The color intensity indicates the relative abundance after *Z*-score normalization. The dendrogram above shows the similarity between samples.

The clustering heatmap generated based on the Top 50 most abundant genera further verified the above results (Figure 4). The three groups of forest musk deer samples clustered in a pattern of within-group aggregation and between-group separation, which was consistent with the results of the *β*-diversity analysis. Samples from the HN captive group independently clustered into a distinct branch, while those from the YS and SX groups clustered together into a larger branch. In the HN captive group, the abundances of genera such as *Parabacteroides*, *Campylobacter*, *Bacteroides*, and *UCG-005* were significantly increased. In the YS wild group, the genera with relatively high abundances included *Novosphingobium*, *Sphingomonas*, *Pedobacter*, and *Devosia*. Although many genera in the SX captive group had similar abundances to those in the YS wild group, the genus *Escherichia-Shigella* exhibited a higher abundance in the SX captive group.

### 3.3 Differential biomarker species analysis

**Figure 5:**
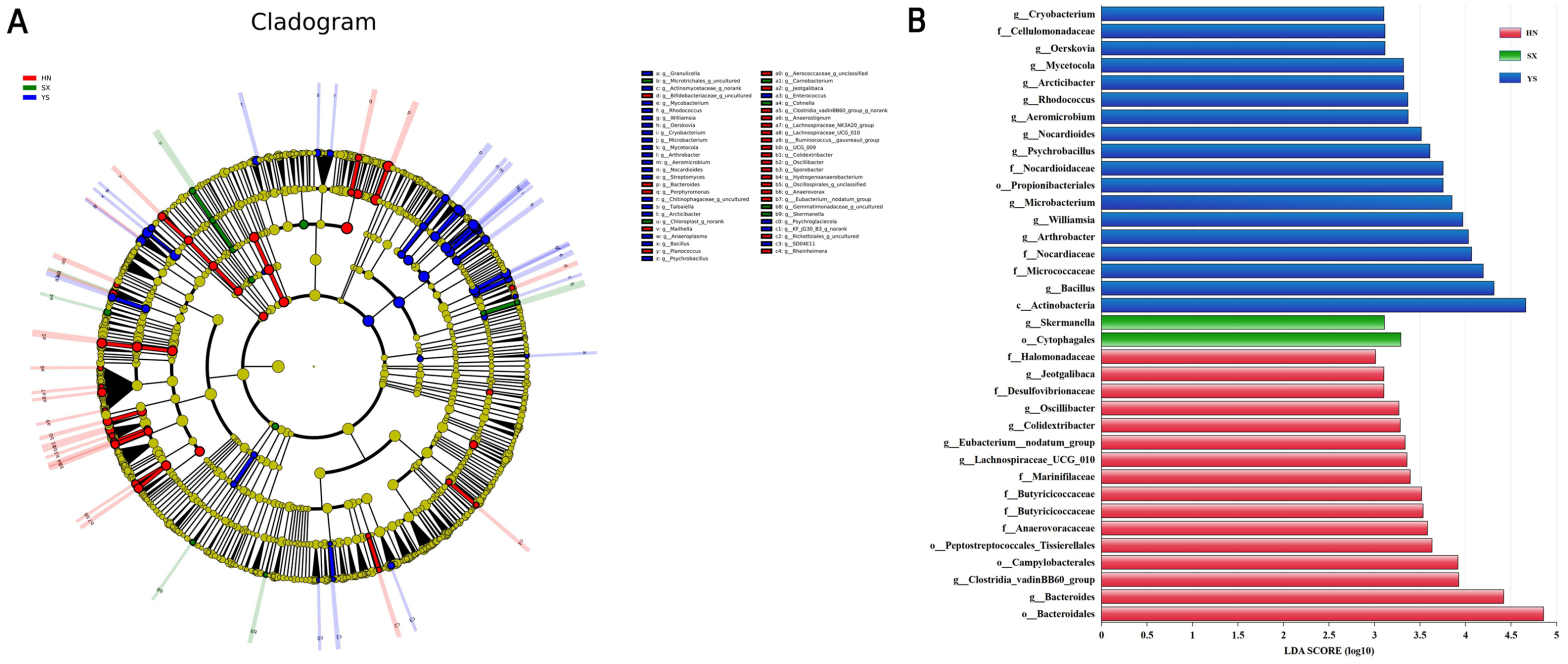
LEfSe analysis of microbiota in feces of three groups of forest musk deer. **(A)** Cladogram. The node size corresponds to the average abundance of taxa, and branches with different colors represent differentially abundant taxa enriched in the corresponding group (red = HN, green = SX, blue = YS). Yellow nodes indicate non-significant taxa. **(B)** LDA score bar chart showing differentially abundant taxa with LDA score > 3. The bar length represents the effect size of differences, and colors correspond to groups.

LEfSe analysis was employed to screen for microbial taxa with significant inter-group differences (LDA score > 2.0). The cladogram (Figure 5A) showed that differential taxa formed distinct evolutionary branches according to the groups: the Actinobacteriota taxa enriched in the YS wild group clustered on one side of the evolutionary tree, while the Bacteroidota and Firmicutes taxa enriched in the HN captive group clustered on the other side. Only a small number of taxa were enriched in the SX captive group. The LDA value bar chart further identified major differential taxa with more prominent effect sizes (LDA score >3.0, Figure 5B), and a total of 36 biomarkers significantly enriched across different groups were identified. The YS wild group was enriched with 18 biomarkers, with Actinobacteriota and its subordinate taxa as the core, including genera such as *Bacillus*, *Arthrobacter*, *Williamsia*, *Microbacterium*, and *Psychrobacillus*, as well as family/order-level taxa such as *Cellulomonadaceae*, *Nocardioidaceae*, *Propionibacteriales*, and *Micrococcaceae*. The HN captive group was enriched with 16 biomarkers, dominated by taxa related to Bacteroidota and Firmicutes, including genera such as *Bacteroides*, *Clostridia*, *Lachnospiraceae_UCG-010*, and *Eubacterium*, as well as family/order-level taxa such as *Anaerovoracaceae*, *Peptostreptococcales-Tissierellales*, *Campylobacterales*, and *Clostridia vadinBB60 group*. The SX captive group was only enriched with 2 taxa, namely *Skermanella* (genus) and *Cytophagales* (order), with the fewest characteristic taxa among the three groups.

### 3.4 Intestinal microbial functional prediction analysis

**Figure 6:**
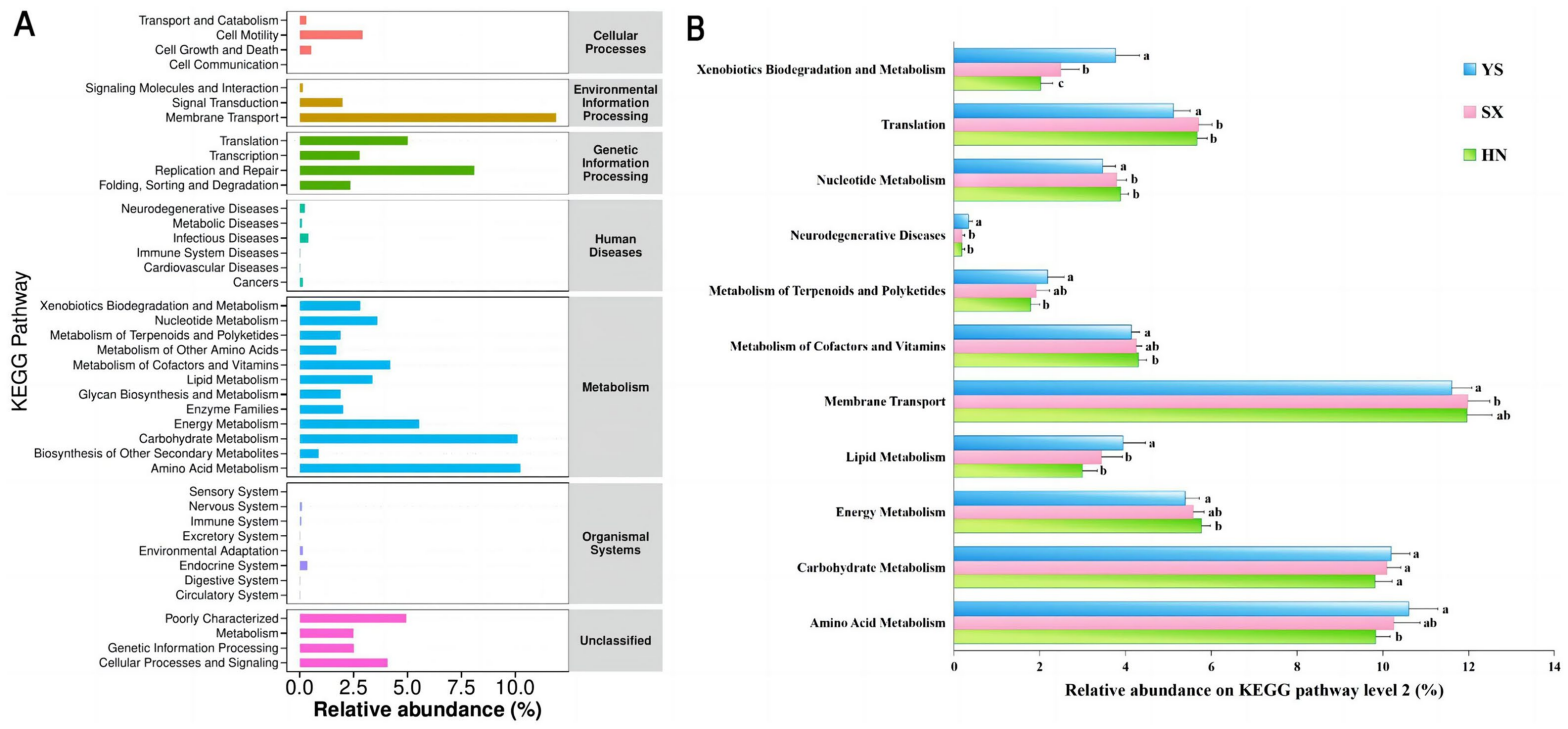
Functional prediction of bacteria based on KEGG database. **(A)** The bar chart illustrates the relative abundance of annotated genes at the level 2 hierarchy of the KEGG database. **(B)** Relative abundance of significantly different KEGG pathways in three groups of forest musk deer. Error bars indicate standard deviation (*n* = 5). Different lowercase letters above bars denote significant differences between groups (Dunn’s *post hoc* test, *p* < 0.05). Shared letters indicate no significant difference.

Functional prediction based on the KEGG database (Figure 6A) showed that the gene functions of the forest musk deer gut microbiota were mainly enriched in seven major categories, among which the metabolism related pathways dominated. The pathways of amino acid metabolism (10.23%) and carbohydrate metabolism (10.09%) had the highest abundance, indicating that the gut microbiota of forest musk deer play important roles in amino acid and carbohydrate metabolism. Notably, the predicted abundance of the membrane transport pathway under the category of environmental information processing reached 11.90%, suggesting that the gut microbiota of forest musk deer may exhibit strong substance transport capacity when adapting to different environmental stresses. These analyses provide functional-level insights into understanding the digestive adaptation mechanism of forest musk deer to high-fiber diets. Further analysis revealed that a total of 11 Level 2 pathways showed significant differences among the three groups (*p* < 0.05) (Figure 6B). Among them, the predicted abundance of the pathways xenobiotics biodegradation and metabolism, lipid metabolism in the YS wild group was significantly higher than that in the two captive groups (*p* < 0.001). Conversely, the two captive groups (HN and SX) showed significantly higher abundance of the pathways translation and nucleotide metabolism compared with the wild group (*p* < 0.01). Between the two captive groups, only the pathway xenobiotics biodegradation and metabolism was significantly higher in the SX group than in the HN group (*p* < 0.05). These results suggest that the gut microbiota of wild forest musk deer may have greater functional potential in xenobiotic detoxification and lipid metabolism, while the microbiota of captive forest musk deer exhibit higher activities of protein synthesis and nucleotide metabolism, indicating significant differences in functional adaptability of the microbiota between the two living environments.

## 4 Discussion

This study systematically compared the gut microbial communities of captive forest musk deer from Henan and Shanxi Provinces with that of wild forest musk deer from Baotianman, Henan Province. The results indicate that a combination of factors—including housing conditions, dietary composition, and geographic location—is associated with the differentiation of forest musk deer gut microbiota. These factors likely interact to shape the microbial characteristics of captive populations.

In terms of community structure, the three groups of forest musk deer all had Firmicutes, Bacteroidota, and Proteobacteria as their dominant microbial phyla, which is consistent with the characteristics of the gut microbiota of most herbivorous mammals (30–32). However, the abundance of Actinobacteriota in the intestinal tract of wild forest musk deer (YS group) was significantly higher than that in the two captive groups. Actinobacteriota can secrete enzyme systems such as cellulases and ligninases to efficiently degrade the diverse high-fiber plants ingested by wild forest musk deer. Meanwhile, the bioactive substances it produces (e.g., antibiotic precursors) can enhance the host’s resistance to the complex wild environment (15). Previous studies have confirmed that genes related to Actinobacteriota are significantly enriched in the metagenome of wild forest musk deer (15, 33), which is consistent with the results of this study and further corroborates the importance of Actinobacteriota for wild forest musk deer to utilize natural food resources. Captive forest musk deer, on the other hand, showed a high abundance of Firmicutes, especially the HN group, where the abundance of Firmicutes reached 59.01%, a phenomenon that is common in captive herbivorous animals (34, 35). The increased ratio of Firmicutes to Bacteroidota is usually associated with increased intake of easily fermentable carbohydrates in the diet. It can produce short-chain fatty acids through the rapid fermentation of non-structural carbohydrates, thereby meeting the host’s energy requirements (36, 37). The abundance of Firmicutes in SX captive group was closer to that of the wild group, which likely reflects the differences in breeding conditions between the two farms. Notably, the abundance of *Escherichia*-*Shigella* in the SX captive group (11.05%) was significantly higher than in the other two groups. While this genus contributes to maintaining gut microbial balance, its overgrowth under captive conditions may be associated with intestinal dysbiosis and an increased risk of digestive disorders (38). Together, these differences likely reflect variations in the dietary composition, housing conditions, and management practices between the two captive populations (39–41).

The gut microbial communities of captive forest musk deer from different geographic origins exhibit significant differentiation. In the SX captive group, the gut microbiota of some individuals clustered with those of the wild group, whereas the HN captive group showed a significant difference from the wild group. It has been reported for Francois’ langurs and Asian black bears that geographic isolation significantly affects the gut microbial community structure by altering vegetation composition and climatic conditions (42, 43). Geographically, Gaoping in Shanxi Province (located on the Loess Plateau, with a temperate continental monsoon climate) and Nanyang in Henan Province (situated in the Nanyang Basin, with a climate transitional from subtropical to warm temperate) differ essentially in climate and native vegetation types. The forage in Gaoping is dominated by the stems and leaves of drought-resistant plants with high lignin content and complex structure (e.g., *Robinia pseudoacacia* leaves, *Ziziphus jujuba* leaves, *Alfalfa* and *Artemisia* species), which are closer to the food resources naturally available to forest musk deer. In contrast, the forage in Nanyang is centered on tender, juicy plants rich in water-soluble fiber, pectin, and starch (e.g., *Mulberry* leaves, *Broussonetia papyrifera* leaves*, Kudzu* leaves and *White clover*), along with a high proportion of concentrate feed, forming a high-energy and easily fermentable dietary system. In this study, the geographical distance between the SX captive group and the YS wild group (approximately 400 km) was greater than that between the HN captive group and the YS wild group (approximately 200 km), yet the microbiota similarity was higher. This indicates that the impact of geographic factors on gut microbiota is exerted through specific environmental variables rather than a simple distance effect. This discovery provides a new approach for the geographic adaptation management of captive forest musk deer that simulating the vegetation composition and environmental conditions of wild habitats, we can promote the convergence of captive gut microbiota toward the wild type, thereby reducing the risk of diseases (44, 45).

In addition to diet, housing conditions may further amplify the microbiota differences: the SX group had a larger activity area with natural soil and vegetation, whereas the HN group was housed in smaller, brick-paved enclosures. Such environmental differences can alter exposure to soil microbes and activity levels, which also influence gut microbiota composition (30). The presence of soil-associated bacteria, particularly *Skermanella*, enriched in the SX captive group warrants explanation. A study on captive red-crowned cranes found that *Skermanella* and *Deinococcus* was enriched after the cranes were placed outdoors and fed fresh fruits and vegetables (46). Nevertheless, other management practices, such as enclosure cleaning frequency and human contact intensity, may also influence gut microbiota by altering environmental microbial exposure or inducing stress responses, though these factors were not quantitatively assessed in the present study. Previous studies have confirmed that season, age, gender, and mating status significantly influence the gut microbial composition of forest musk deer (47–49). In our study, all samples were collected in April (spring), which partially controls for seasonal variation. However, differences in age and gender composition among groups, as well as unknown mating status, may still act as potential confounding factors influencing gut microbial variation.

KEGG notation revealed that the gut microbiota of forest musk deer are predominantly enriched in major functional categories including metabolism and environmental information processing. Key pathways identified were amino acid metabolism (10.23%), carbohydrate metabolism (10.09%), and membrane transport (11.90%), suggesting a potential role of gut microbiota in nutrient metabolism and absorption (14, 15). However, the differences in microbial community structure directly lead to adaptive differentiation at the functional level (50). The YS wild group exhibited significantly higher abundance in xenobiotics biodegradation and metabolism pathways compared to the captive group, which is closely related to the secondary metabolites such as phenols and terpenoids in the plants they consume (51). The gut microbiota detoxify or transform these substances through specific enzyme systems, reducing the host’s risk of poisoning (52, 53). The higher predicted abundance of lipid metabolism pathways in the wild YS group could be related to their natural foraging behavior and possibly to musk secretion activity. Musk secretion is a lipid-rich process requiring substantial energy and precursor metabolites. Recent studies (54, 55) have shown that gut microbiota-derived short-chain fatty acids and lipid metabolites may influence musk biosynthesis.

Since we lack individual-level musk secretion data for the wild samples, we cannot exclude the possibility that some samples were collected from male deer during the pre-musk secretion period. In contrast, captive forest musk deer showed higher abundance of pathways related to translation and nucleotide metabolism, which may contribute to efficient protein synthesis and cell proliferation under stable feeding conditions (15, 56). The abundance of xenobiotics biodegradation pathway in the SX captive group was significantly higher than that in the HN captive group, which may reflect dietary and environmental differences between the two captive populations. These observations suggest that optimizing diet composition and semi-natural housing conditions may help modulate the gut microbiota function of captive forest musk deer (32, 44, 57). Combined with the *α*-diversity results, although there was no significant difference among the three groups, the *β*-diversity and functional pathway differences were significant. This indicates that the microbial community of structural differentiation precedes diversity changes, which provides an important warning for captive species conservation: even if microbial diversity does not decline, the cryptic differentiation of structure and function may still threaten host health, thus, strengthening the monitoring of microbial community structure is necessary.

Nevertheless, this study has several limitations. The small sample size may limit the generalizability of our conclusions, and the lack of molecular individual identification for wild samples and incomplete information on mating status and musk secretion status represent additional constraints. Furthermore, our study design does not allow fully disentangling the relative contributions of diet, housing conditions, and geographic factors, and validated gut health indices (e.g., GMHI/MDI) were not applied due to the absence of species-specific healthy reference standards for forest musk deer. Future studies with larger sample sizes and integrated multidimensional ecological data are needed to further validate and expand our findings.

## 5 Conclusion

The gut microbiota of wild forest musk deer is characterized by high abundance of the phylum Actinobacteriota and enrichment of functions related to xenobiotic degradation and lipid metabolism, whereas captive forest musk deer exhibit high abundance of the phylum Firmicutes and active translation and nucleotide metabolism. A combination of factors—including dietary composition, housing conditions, and geographic location—likely influences the microbial community structure of captive populations. A stable core microbiota exists in the forest musk deer’s gut, which provides the basis for maintaining basic intestinal functions.

## Data Availability

The original contributions presented in the study are publicly available. This data can be found here: NCBI, accession number PRJNA1468187.

## References

[1] Wang Y Harris R. *Moschus berezovskii* (errata version published in 2016). *The IUCN red list of threatened species* (2015):e.T13894A103431781. doi: 10.2305/IUCN.UK.2015-4.RLTS.T13894A61976926.en,

[2] Wang YL Ha CY. Research progress on musk and artificial propagation technique of forest musk deer (*Moschus berezovskii*). Zhongguo Zhong Yao Za Zhi. (2018) 43:3806–10. doi: 10.19540/j.cnki.cjcmm.20180726.004, 30453702

[3] Li Y Zhang T Shi M Zhang B Hu X Xu S . Characterization of intestinal microbiota and fecal cortisol, T3, and IgA in forest musk deer (*Moschus berezovskii*) from birth to weaning. Integr Zool. (2021) 16:33452844:300–12. doi: 10.1111/1749-4877.12522 PMC8248411 33452844

[4] Yang C Huang W Sun Y You L Jin H Sun Z. Effect of probiotics on diversity and function of gut microbiota in *Moschus berezovskii*. Arch Microbiol. (2021) 203:3305–15. doi: 10.1007/s00203-021-02315-5, 33860850

[5] de Vos WM Tilg H Van Hul M Cani PD. Gut microbiome and health: mechanistic insights. Gut. (2022) 71:1020–32. doi: 10.1136/gutjnl-2021-326789, 35105664 PMC8995832

[6] D'Aquila P Carelli LL De Rango F Passarino G Bellizzi D. Gut microbiota as important mediator between diet and DNA methylation and histone modifications in the host. Nutrients. (2020) 12:597. doi: 10.3390/nu12030597, 32106534 PMC7146473

[7] Li N Guo X. The gut microbiota and host immunity synergistically orchestrate colonization resistance. Gut Microbes. (2026) 18:2611545. doi: 10.1080/19490976.2025.2611545, 41482864 PMC12773493

[8] Ying Z Yang Q Xie S Cai M Fan W Gao H . Active dry yeast enhances immunity through modulation of gut microbiota and serum metabolic processes in captive forest musk deer (*Moschus berezovskii*). BMC Vet Res. (2025) 21:262. doi: 10.1186/s12917-025-04705-z, 40221712 PMC11992737

[9] Dai Q Wu J Chen F Jiang G. Fecal steroids, short-chain fatty acids, and microbiota in high- versus low-yielding forest musk deer (*Moschus berezovskii*). AMB Express. (2025) 15:168. doi: 10.1186/s13568-025-01967-6, 41212453 PMC12602856

[10] Su R Dalai M Luvsantseren B Chimedtseren C Hasi S. Comparative study of the function and structure of the gut microbiota in Siberian musk deer and forest musk deer (*Moschus berezovskii*). Appl Microbiol Biotechnol. (2022) 106:6799–817. doi: 10.1007/s00253-022-12158-9, 36100751

[11] Guo W Mishra S Wang C Zhang H Ning R Kong F . Comparative study of gut microbiota in wild and captive giant pandas (*Ailuropoda melanoleuca*). Genes. (2019) 10:827. doi: 10.3390/genes10100827, 31635158 PMC6826394

[12] Huang X Li H Zhang L Zhang X Cheng S Yan Y . Comparative analysis of gut microbiota between wild and captive Guizhou snub-nosed monkey (*Rhinopithecus brelichi*). Ecol Evol. (2024) 14:e70690. doi: 10.1002/ece3.70690, 39664719 PMC11631709

[13] Hu X Liu G Li Y Wei Y Lin S Liu S . High-throughput analysis reveals seasonal variation of the gut microbiota composition within forest musk deer (*Moschus berezovskii*). Front Microbiol. (2018) 9:1674. doi: 10.3389/fmicb.2018.01674, 30093891 PMC6070636

[14] Jiang F Gao H Qin W Song P Wang H Zhang J . Marked seasonal variation in structure and function of gut microbiota in forest and alpine musk deer (*Moschus berezovskii*). Front Microbiol. (2021) 12:699797. doi: 10.3389/fmicb.2021.699797 34552569 PMC8450597

[15] Liu H Xiao L Liu Z Deng Y Zhu J Yang C . Impacts of captive domestication and geographical divergence on the gut microbiome of endangered forest musk deer (*Moschus berezovskii*). Animals. (2025) 15:1954. doi: 10.3390/ani15131954 40646853 PMC12248866

[16] Ross FC Patangia D Grimaud G Lavelle A Dempsey EM Ross RP . The interplay between diet and the gut microbiome: implications for health and disease. Nat Rev Microbiol. (2024) 22:671–86. doi: 10.1038/s41579-024-01068-4, 39009882

[17] Yuan NX Qin YH Wang J Shen LQ Gao HX Xiang RW . Musk secretion of endangered alpine musk deer (*Moschus chrysogaster*): muscone content and the relationships to age, health, mating history and enclosure condition. Biologia. (2021) 76:3761–7. doi: 10.1007/s11756-021-00879-7

[18] Lu X Sheng Y Ye H Yang Z Meng X. Development of stereotypic behaviors and personality traits of captive male Forest musk deer and relationships with musk secretion. Vet Sci. (2026) 13:261. doi: 10.3390/vetsci13030261, 41893678 PMC13030802

[19] Dai T Wen D Bates CT Wu L Guo X Liu S . Nutrient supply controls the linkage between species abundance and ecological interactions in marine bacterial communities. Nat Commun. (2022) 13:175. doi: 10.1038/s41467-021-27857-6, 35013303 PMC8748817

[20] Caporaso JG Lauber CL Walters WA Berg-Lyons D Lozupone CA Turnbaugh PJ . Global patterns of 16S rRNA diversity at a depth of millions of sequences per sample. Proc Natl Acad Sci USA. (2011) 108:4516–22. doi: 10.1073/pnas.1000080107, 20534432 PMC3063599

[21] Bolger AM Lohse M Usadel B. Trimmomatic: a flexible trimmer for Illumina sequence data. Bioinformatics. (2014) 30:2114–20. doi: 10.1093/bioinformatics/btu170, 24695404 PMC4103590

[22] Edgar RC Haas BJ Clemente JC Quince C Knight R. UCHIME improves sensitivity and speed of chimera detection. Bioinformatics. (2011) 27:2194–200. doi: 10.1093/bioinformatics/btr381, 21700674 PMC3150044

[23] Caporaso JG Kuczynski J Stombaugh J Bittinger K Bushman FD Costello EK . QIIME allows analysis of high-throughput community sequencing data. Nat Methods. (2010) 7:335–6. doi: 10.1038/nmeth.f.303, 20383131 PMC3156573

[24] Wang Q Garrity GM Tiedje JM Cole JR. Naive Bayesian classifier for rapid assignment of rRNA sequences into the new bacterial taxonomy. Appl Environ Microbiol. (2007) 73:5261–7. doi: 10.1128/AEM.00062-07, 17586664 PMC1950982

[25] Schloss PD Westcott SL Ryabin T Hall JR Hartmann M Hollister EB . Introducing mothur: open-source, platform-independent, community-supported software for describing and comparing microbial communities. Appl Environ Microbiol. (2009) 75:7537–41. doi: 10.1128/AEM.01541-09, 19801464 PMC2786419

[26] Segata N Izard J Waldron L Gevers D Miropolsky L Garrett WS . Metagenomic biomarker discovery and explanation. Genome Biol. (2011) 12:R60. doi: 10.1186/gb-2011-12-6-r60, 21702898 PMC3218848

[27] Li J Mavrodi OV Hou J Blackmon C Babiker EM Mavrodi DV. Comparative analysis of rhizosphere microbiomes of southern highbush blueberry (*Vaccinium corymbosum*), Darrow’s blueberry (*Vaccinium darrowii*), and Rabbiteye blueberry (*Vaccinium virgatum*). Front Microbiol. (2020) 11:370. doi: 10.3389/fmicb.2020.00370, 32226421 PMC7081068

[28] Zou Y Zou Q Yang H Han C. Investigation of intestinal microbes of five zokor species based on 16S rRNA sequences. Microorganisms. (2024) 13:27. doi: 10.3390/microorganisms13010027, 39858794 PMC11767591

[29] Langille MG Zaneveld J Caporaso JG McDonald D Knights D Reyes JA . Predictive functional profiling of microbial communities using 16S rRNA marker gene sequences. Nat Biotechnol. (2013) 31:814–21. doi: 10.1038/nbt.2676, 23975157 PMC3819121

[30] Qin W Song P Zhang S. Seasonal and soil microbiota effects on the adaptive strategies of wild goitered gazelles based on the gut microbiota. Front Microbiol. (2022) 13:918090. doi: 10.3389/fmicb.2022.918090, 35859737 PMC9289685

[31] Chen S Holyoak M Liu H Bao H Ma Y Dou H . Effects of spatially heterogeneous warming on gut microbiota, nutrition and gene flow of a heat-sensitive ungulate population. Sci Total Environ. (2022) 806:150537. doi: 10.1016/j.scitotenv.2021.150537, 34844317

[32] Wang Y Xu B Chen H Yang F Huang J Jiao X . Environmental factors and gut microbiota: toward better conservation of deer species. Front Microbiol. (2023) 14:1136413. doi: 10.3389/fmicb.2023.1136413, 36960286 PMC10027939

[33] Zhang B Shi M Xu S Zhang H Li Y Hu D. Analysis on changes and influencing factors of the intestinal microbiota of alpine musk deer between the place of origin and migration. Animals. (2023) 13:3791. doi: 10.3390/ani13243791, 38136828 PMC10740494

[34] de Oliveira MN Jewell KA Freitas FS Benjamin LA Tótola MR Borges AC . Characterizing the microbiota across the gastrointestinal tract of a Brazilian Nelore steer. Vet Microbiol. (2013) 164:307–14. doi: 10.1016/j.vetmic.2013.02.013 23490556

[35] Wang L Jin L Xue B Wang Z Peng Q. Characterizing the bacterial community across the gastrointestinal tract of goats: composition and potential function. Microbiology. (2019) 8:e00820. doi: 10.1002/mbo3.820, 30829000 PMC6741129

[36] Xue B Xie J Huang J Chen L Gao L Ou S . Plant polyphenols alter a pathway of energy metabolism by inhibiting fecal Bacteroidetes and Firmicutes *in vitro*. Food Funct. (2016) 7:1501–7. doi: 10.1039/c5fo01438g, 26882962

[37] Salamone D Rivellese AA Vetrani C. The relationship between gut microbiota, short-chain fatty acids and type 2 diabetes mellitus: the possible role of dietary fibre. Acta Diabetol. (2021) 58:1131–8. doi: 10.1007/s00592-021-01727-5, 33970303 PMC8316221

[38] Li HZ Li N Wang JJ Li H Huang X Guo L . Dysbiosis of gut microbiome affecting small intestine morphology and immune balance: a rhesus macaque (*Macaca mulatta*) model. Zool Res. (2020) 41:20–31. doi: 10.24272/j.issn.2095-8137.2020.004 31930784 PMC6956715

[39] Milani C Alessandri G Mancabelli L Mangifesta M Lugli GA Viappiani A . Multi-omics approaches to decipher the impact of diet and host physiology on the mammalian gut microbiome. Appl Environ Microbiol. (2020) 86:e01864–20. doi: 10.1128/AEM.01864-20, 32948523 PMC7657629

[40] Tsukayama P Boolchandani M Patel S Pehrsson EC Gibson MK Chiou KL . Characterization of wild and captive baboon gut microbiota and their antibiotic resistomes. mSystems. (2018) 3:e00016-18. doi: 10.1128/mSystems.00016-18, 29963641 PMC6020475

[41] Martínez-Mota R Kohl KD Orr TJ Dearing MD. Natural diets promote retention of the native gut microbiota in captive rodents. ISME J. (2020) 14:67–78. doi: 10.1038/s41396-019-0497-6, 31495829 PMC6908644

[42] Sun Y Yu Y Wu A Zhang C Liu X Qian C . The composition and function of the gut microbiota of Francois' langurs (*Trachypithecus francoisi*) depend on the environment and diet. Front Microbiol. (2023) 14:1269492. doi: 10.3389/fmicb.2023.1269492, 38033571 PMC10687571

[43] Song C Wang B Tan J Zhu L Lou D Cen X. Comparative analysis of the gut microbiota of black bears (*Ursus thibetanus*) in China using high-throughput sequencing. Mol Gen Genomics. (2017) 292:407–14. doi: 10.1007/s00438-016-1282-0, 28028611

[44] Dallas JW Warne RW. Captivity and animal microbiomes: potential roles of microbiota for influencing animal conservation. Microb Ecol. (2023) 85:820–38. doi: 10.1007/s00248-022-01991-0, 35316343

[45] Tang S Li Y Huang C Yan S Li Y Chen Z . Comparison of gut microbiota diversity between captive and wild tokay gecko (*Gekko gecko*). Front Microbiol. (2022) 13:897923. doi: 10.3389/fmicb.2022.897923, 35783386 PMC9248866

[46] Xu W Xu N Zhang Q Tang K Zhu Y Chen R . Association between diet and the gut microbiome of young captive red-crowned cranes (*Grus japonensis*). BMC Vet Res. (2023) 19:80. doi: 10.1186/s12917-023-03636-x, 37391732 PMC10311889

[47] Xu Z Li F Liu Q Ma T Feng X Zhao G . Chemical composition and microbiota changes across musk secretion stages of forest musk deer (*Moschus berezovskii*). Front Microbiol. (2024) 15:1322316. doi: 10.3389/fmicb.2024.1322316 38505545 PMC10948612

[48] Li D Chen B Zhang L Gaur U Ma T Jie H . The musk chemical composition and microbiota of Chinese forest musk deer males. Sci Rep. (2016) 6:18975. doi: 10.1038/srep18975, 26744067 PMC4705530

[49] Zhao G Ma T Tang W Li D Mishra SK Xu Z . Gut microbiome of Chinese Forest musk deer examined across gender and age. Biomed Res Int. (2019) 2019:1–10. doi: 10.1155/2019/9291216, 31886268 PMC6925676

[50] Ning Y Qi J Dobbins MT Liang X Wang J Chen S . Comparative analysis of microbial community structure and function in the gut of wild and captive Amur tiger (*Panthera tigris altaica*). Front Microbiol. (2020) 11:1665. doi: 10.3389/fmicb.2020.01665 32793154 PMC7393233

[51] Tan Y An K Su J. Review: mechanism of herbivores synergistically metabolizing toxic plants through liver and intestinal microbiota. Comp Biochem Physiol C Toxicol Pharmacol. (2024) 281:109925. doi: 10.1016/j.cbpc.2024.109925, 38643812

[52] Dearing MD Weinstein SB. Metabolic enabling and detoxification by mammalian gut microbes. Ann Rev Microbiol. (2022) 76:579–96. doi: 10.1146/annurev-micro-111121-085333, 35671535

[53] Kohl KD Dearing MD. The woodrat gut microbiota as an experimental system for understanding microbial metabolism of dietary toxins. Front Microbiol. (2016) 7:1165. doi: 10.3389/fmicb.2016.01165, 27516760 PMC4963388

[54] Wang T Yang M Shi X Tian S Li Y Xie W . Multiomics analysis provides insights into musk secretion in muskrat and musk deer. Gigascience. (2025) 14:giaf006. doi: 10.1093/gigascience/giaf006, 40036429 PMC11878540

[55] Sun YS Zhao L Zheng CL Yan XT Li Y Gao XL . Convergent musk biosynthesis across host and microbiota in musk deer and muskrat. Zool Res. (2025) 46:505–17. doi: 10.24272/j.issn.2095-8137.2025.094, 40259731 PMC12361909

[56] Zhang K Wang X Gong X Sui J. Gut microbiome differences in rescued common kestrels (*Falco tinnunculus*) before and after captivity. Front Microbiol. (2022) 13:858592. doi: 10.3389/fmicb.2022.858592, 35794924 PMC9251364

[57] Wang H Ali M Zhu Y Chen X Lu D Liu Y . Comparative analysis of gut microbiota in free range and house fed yaks from Linzhou County. Sci Rep. (2025) 15:14317. doi: 10.1038/s41598-025-95357-4, 40274860 PMC12022119

