# Supplementary material for: Differential analysis of gut microbiota between captive and wild forest musk deer (Moschus berezovskii) based on 16S rRNA sequencing
Source: Front Vet Sci. 2026 Jun 4;13:1824527. doi: 10.3389/fvets.2026.1824527 (PMC13275715; doi:10.3389/fvets.2026.1824527)
Supplement: Supplementary file 1 [file Data_Sheet_1.PDF]

Supplementary Material

1 Supplementary Tables

Supplementary Table S1. Sample Information (HN1-HN5: HN group; SX1-SX5: SX group; YS1-YS5: YS group）

Sample ID	Collection Location	gender	Age (year)	Latitude and longitude coordinates	Altitude /m	Primary Food Source
HN01	Xixia County, Nanyang City, Henan Province, China	male	1	33°21′N 111°25′E	292	*Mulberry* leaves, *Broussonetia papyrifera* leaves, *Kudzu* leaves, and *White clover*, etc. Supplementary feed: Pumpkin, carrot and concentrated feed.
HN02		male	1			
HN03		male	3			
HN04		female	1			
HN05		female	1			
SX01	Gaoping City, Shanxi Province, China	female	3	35°45′N, 113°5′E	957	*Mulberry leaves, Robinia pseudoacacia* leaves, *Ziziphus jujuba* leaves and *Artemisia* species, etc. Supplementary feed: Pumpkin, carrot and concentrated feed.
SX02		female	3			
SX03		female	1			
SX04		male	1			
SX05		female	3			
YS01	Neixiang County, Nanyang City, Henan Province, China	-	-	33°20′～33°36′N, 111°47′～112°04′E,	1200–1500	Leaves of broad-leaved trees such as *Quercus aliena* and *Betula platyphylla*; *Taraxacum mongolicum*, *Viola philippica*, etc.
YS02		-	-			
YS03		-	-			
YS04		-	-			
YS05		-	-			

Supplementary Table S2. Statistics of 16S rRNA Sequencing Data Information. Raw Reads: Original offline data; Average Length: the average sequence length of optimized data; Q30: The proportion of bases with a quality value of 30 or more; Q20: The proportion of bases with a quality value of 20 or more.

Sample	Raw Reads	Valid Reads	Average Length	Valid Bases	Q30（%）	Q20（%）
HN01	96156	95702	413	39524776	94.64	98.31
HN02	96061	95570	422.1	40343505	94.25	98.15
HN03	94549	94050	414.8	39007672	94.63	98.3
HN04	95433	94926	412.1	39116760	94.43	98.24
HN05	93388	92980	411.1	38222911	94.68	98.34
SX01	96650	96070	416.8	40038989	94.55	98.27
SX02	93911	93433	418.4	39088160	94.81	98.37
SX03	97904	97404	412.5	40181025	94.58	98.3
SX04	95375	94827	417.2	39561446	94.4	98.21
SX05	94751	94341	418.2	39456495	94.31	98.17
YS01	98093	97642	414.5	40468724	94.5	98.25
YS02	93392	92856	418.5	38863541	94.46	98.21
YS03	93112	92691	414.8	38444329	94.89	98.4
YS04	93548	93090	415.6	38686313	94.44	98.2
YS05	97957	97429	415	40431126	94.5	98.23

Supplementary Table S3. Statistical Table of α-Diversity Index

Sample ID	Reads	OTU	ace	chao	coverage	shannon	simpson
HN01	91287	999	1112	1148	0.998116	4.57	0.0272
HN02	90969	1066	1205	1190	0.997911	4.3	0.0435
HN03	88531	1134	1315	1313	0.997526	5.1	0.0174
HN04	88716	1229	1370	1378	0.997734	5.01	0.0218
HN05	87594	1175	1343	1348	0.997545	4.96	0.0187
SX01	90944	1201	1388	1381	0.997449	4.59	0.0595
SX02	88895	1269	1477	1466	0.997098	4.18	0.1046
SX03	91202	1396	1595	1614	0.997138	5.35	0.0106
SX04	89942	1086	1297	1286	0.997232	4.6	0.0245
SX05	88911	953	1203	1191	0.997042	4.34	0.0295
YS01	91611	1173	1321	1312	0.997806	5.09	0.0178
YS02	88557	884	1134	1112	0.997154	4.13	0.0381
YS03	88037	939	1127	1107	0.997512	4.42	0.0306
YS04	88201	1227	1464	1443	0.996961	4.87	0.0192
YS05	90524	1153	1322	1330	0.997570	4.75	0.0337

2 Supplementary Figures

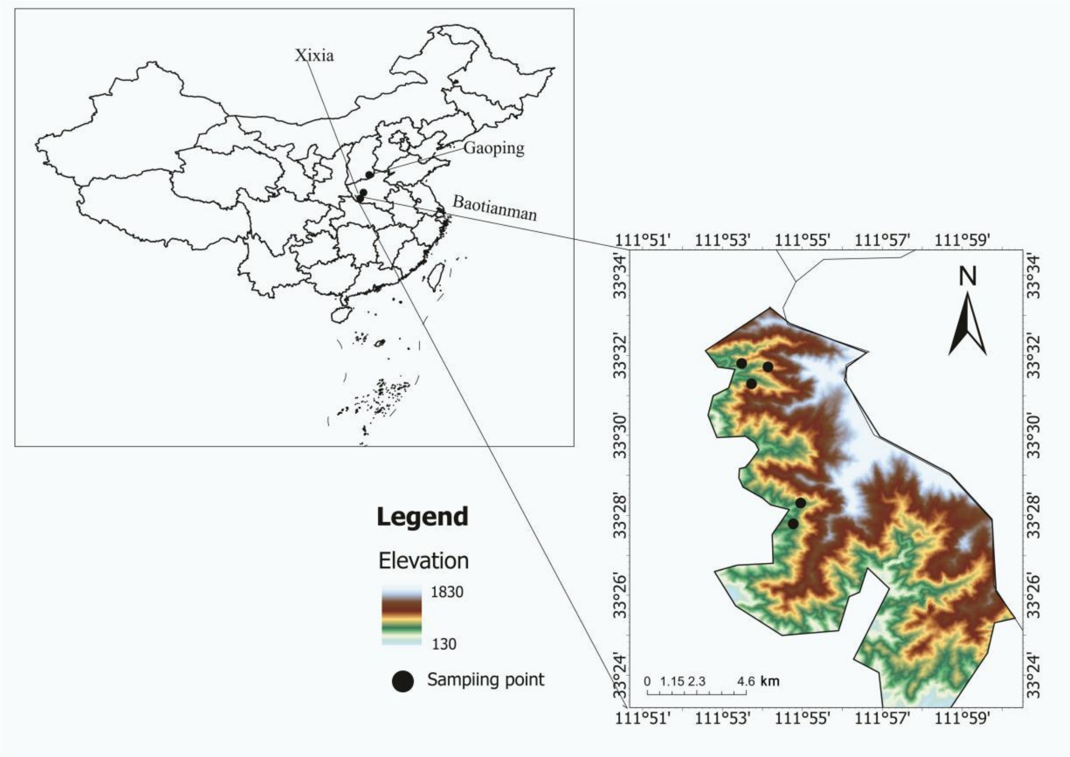

Supplementary Figure S1. Sampling sites of captive and wild forest musk deer.

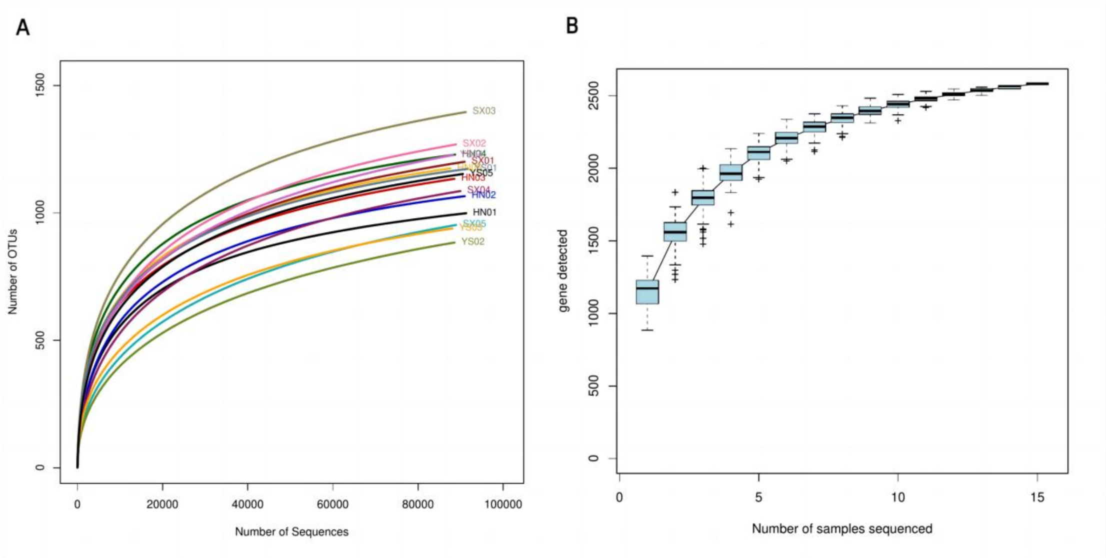

Supplementary Figure S2. Evaluation of sequencing depth for forest musk deer fecal samples. (A)Rarefaction curve; (B) species accumulation curves.
